# Chemical bonding and electronic properties along Group 13 metal oxides

**DOI:** 10.1007/s00894-024-05957-6

**Published:** 2024-05-07

**Authors:** Samadhan Kapse, Maria Voccia, Francesc Viñes, Francesc Illas

**Affiliations:** Departament de Ciència de Materials i Química Física & Institut de Química Teòrica i Computacional (IQTCUB), Universitat de Barcelona, C/Martí I Franquès 1-11, 08028 Barcelona, Spain

**Keywords:** Ionicity, Catalyst, Metal oxides, DFT, Bader charge

## Abstract

**Context:**

The present work provides a systematic theoretical analysis of the nature of the chemical bond in Al_2_O_3_, Ga_2_O_3_, and In_2_O_3_ group 13 cubic crystal structure metal oxides. The influence of the functional in the resulting band gap is assessed. The topological analysis of the electron density provides unambiguous information about the degree of ionicity along the group which is linearly correlated with the band gap values and with the cost of forming a single oxygen vacancy. Overall, this study offers a comprehensive insight into the electronic structure of metal oxides and their interrelations. This will help researchers to harness information effectively, boosting the development of novel metal oxide catalysts or innovative methodologies for their preparation.

**Methods:**

Periodic density functional theory was used to predict the atomic structure of the materials of interest. Structure optimization was carried out using the PBE functional, using a plane wave basis set and the PAW representation of the atomic cores, using the VASP code. Next, the electronic properties were computed by carrying out single point calculations employing PBE, PBE + U functionals using VASP and also with PBE and the hybrid HSE06 functionals using the FHI-AIMS software. For the hybrid HSE06, the impact of the screening parameter, ω, and mixing parameter, *α*, on the calculated band gap has also been assessed.

**Supplementary Information:**

The online version contains supplementary material available at 10.1007/s00894-024-05957-6.

## Introduction

Metal oxide catalysts have become fundamental elements in a multitude of catalytic reactions due to their versatile properties and extensive applicability [1–5]. Their integration into industrial processes traces back to the mid-1950s, a period characterized by their effective utilization in a variety of oxidation reactions, particularly prominent in hydrocarbon processing. Metal oxides, categorized within the realm of inorganic materials, showcase a diverse array of unique properties and functionalities, endowing them with indispensable roles across various sectors including sensing technologies, catalysis, and energy conversion systems such as fuel cells, to name a few [6, 7]. Metal oxides, characterized by metal–oxygen bonds, constituting fundamental repeating units, serve as catalysts with pertinent surface, morphological, and solid-state attributes, pivotal for the execution of intricate heterogeneous catalytic reactions. A meticulous examination of the interconnections and correlations between the physical attributes and the catalytic activity of these metal oxide catalysts is imperative to foster the development of environmentally friendly, enduring, potent, and selective catalysts. The diversity of metal oxides manifests in distinct structural compositions and properties such as simple oxides or transition metal oxides, each family with well-defined physical and chemical properties [8–10].

Metal oxides find extensive advantages in catalysis owing to their inherent resistance to poisoning. The metal oxides also serve as ideal supports for the synthesis of catalysts containing noble metals [11, 12]. The classification of metal oxides into single or mixed oxides is contingent upon the number of metal cations within their structure. The immobilization of a catalyst assumes a critical role in augmenting both its surface area and longevity, serving as primary functions of metal oxide catalytic supports. Essentially, metal oxides are commonly employed as supports for noble metal catalysts, particularly nanoparticles. These supports may also function as co-catalysts, enhancing overall catalytic activity based on the nature of the catalyzed reaction. Furthermore, surface modification of metal oxides with base or noble metal nanoparticles is essential to reduce catalytic reaction barrier and enhancing gas sensing performance [13, 14]. Various industries, notably the automotive sector, adopt metal oxide materials, particularly in applications like gas purification, where the size of the metal oxide-supported noble metal significantly influences effectiveness [15]. Metal oxides proved to be the most economically efficient option with lower toxicity levels compared to zeolites and MOFs within the realm of absorbents. Currently, oxide-based sorbents exhibit high selectivity in capturing carbon dioxide under a wide range of pressure and temperature conditions [16–18].

The catalytic interest on Group 13 oxides is gaining momentum. For instance, aluminum oxide (Al_2_O_3_) stands out as the optimal catalyst support among metal oxides due to its affordability, substantial and thermally stable surface area, controllable porosity, and inherent resistance to steam [19, 20]. Recently, indium oxide (In_2_O_3_)-based catalysts gained high attention due to the superior activity towards CO_2_ conversion and excellent selectivity to methanol even at high temperature [21, 22]. Similarly, gallium oxide (Ga_2_O_3_)-containing catalysts have been investigated for alkane oxidative dehydrogenation processes [23, 24].

The properties of metal oxides are defined by the nature of the chemical bonding which, in turn, is heavily dominated by the ionicity. This is because the electrostatic contribution from the Madelung potential is a key ingredient of the stability of these systems. In the case of alkaline-earth oxides, ab initio Hartree–Fock and configuration interaction calculations on embedded cluster models indicate that the nature of the chemical bond is almost full ionic along the series with net charges close to the ones expected from the formal oxidation state [25, 26]. For corundum, the most stable polymorph of Al_2_O_3_, there is also evidence of an almost fully ionic picture [26, 27], and this also the case for rock-salt (TiO) titanium oxide, and corundum-like polymorph (Ti_2_O_3_), although the ionic picture breaks down for rutile (TiO_2_) because of the excessive cost to generate the highly charged Ti^+4^ cations [28]. However, the information regarding the chemical bonding in the rest of Group 13 oxides is scarce and urgently needed to develop different applications based on these materials including electronics and catalysis. To provide a comprehensive picture of chemical bonding and electronic structure of these metal oxides, we carried out a systematic research based on state-of-the-art periodic density functional theory (DFT) calculations. The reported results on the trend of electronic properties of these materials are expected to contribute to better understand the catalytic reactivity of these metal oxides, which can strengthen the pathway towards the development of improved performance catalysts.

### Theoretical methodology and computational details

To analyze the trends in chemical bonding along the Al_2_O_3_, Ga_2_O_3_, and In_2_O_3_ series, we considered the experimentally stable cubic polymorph of these oxides [29, 30]. For comparison, we included magnesium oxide (MgO) [31] a material for which there is broad consensus that chemical bonding is almost purely ionic. The initial model structures are taken from the inorganic crystal structure database (ICSD) uploaded on the Materials Project webpage [32]. The cubic bixbyite-type phase for Al_2_O_3_, Ga_2_O_3_, and In_2_O_3_ correspond to the Iā3 space group and contains a total of 80 atoms, 32 metal, and 48 oxygen. For MgO, we considered the rock-salt cubic unit cell with space group Fm̅3m containing eight atoms (four Mg and four O).

To analyze the chemical bond in these materials, first principles DFT calculations were conducted using the Vienna ab initio simulation package (VASP) [33]. In an initial step, the atomic structure was optimized using the Perdew-Burke-Ernzerhof (PBE) [34] functional within the generalized gradient approximation (GGA). The Kohn–Sham equations were iteratively solved until self-consistency by expanding the valence electron density in a plane-wave basis set with a cut-off energy of 415 eV, while the interaction between valence electrons and atomic cores was accounted for by means of the projector-augmented wave (PAW) method [35, 36]. More precisely, the number of electrons explicitly accounted for is six for Mg, thirteen for Al, Ga, and In, and eight for O.

Convergence thresholds were set at 10^−5^ eV for total energies and 0.01 eV/Å for forces. All calculations were carried out in a non-spin-polarized fashion since the materials of interest are non-magnetic metal oxides. After performing a pertinent **k**-point density convergence test with total energy converged up to 1 meV, the Brillouin zone of MgO was sampled with a 9 × 9 × 9 grid of Monkhorst–Pack special **k**-points [37], whereas a 3 × 3 × 3 grid was used for the Group 13 oxides. For all oxides, atoms and cells are fully allowed to relax towards converging optimized crystal structure. Further, denser Monkhorst–Pack grids of 17 × 17 × 17 for MgO and 9 × 9 × 9 for other oxides were used to accurately describe the electronic structure at the optimized structures. The followed path along the reducible Brillouin zone is shown in Figure S1 in the Supporting Information (SI).

Given that GGA functionals tend to underestimate the binding energy associated with *d* electrons, thereby resulting in a significant overestimation of the *p*–*d* hybridization in oxides [38], and a considerable underestimation of the band gap, it is necessary to go beyond the semi-local nature of this functional [39, 40]. To mitigate this issue and facilitate the localization of *d* electrons, one can rely on hybrid functionals including a part of non-local Fock exchanges, as done in the widely used PBE0 and Heyd-Scuseria-Ernzerhof (HSE06) functionals [41, 42]. Nevertheless, one must advert that the amount of Fock exchange required to reproduce the experimental gap of these materials may vary from oxide to oxide; e.g. 35% for NiO [40], 25% for ZnO [43], and 12.5% for TiO_2_ rutile and anatase [44]. Dielectric-dependent functionals offer some advantages as reproduce quite well experimental band gaps with notable exceptions [45], but at the cost of introducing one parameter, either experimental or calculated, which is external to the theory as it does not enter in the Hamiltonian of the system.

In a first step, the electronic structure of these materials was studied using the on-site Hubbard-like *U* parameter introduced to avoids an excessive delocalization of the d electrons in transition metal oxides [46]. Based on previous works [38, 47], a value* U*= 7 eV is adopted for all *np* levels of these oxides. Next, we performed periodic DFT calculations with the hybrid HSE06 functionals to calculate electronic properties of metal oxides using the PBE optimized structure with VASP. To avoid the high computational cost of hybrid calculations with a plane wave basis set, this additional set of calculations was carried out in a single point fashion using the Fritz-Haber institute ab initio materials simulations (FHI-AIMS) code which explicitly incorporates all electrons and uses numerical atom-centred orbitals (NAO) to describe the electron density [48, 49]. A light grid, Tier-1 basis set was used, with **k**-points Monkhorst–Pack meshes of 9 × 9 × 9 for MgO and 3 × 3 × 3 for the other oxides. For calculations with the hybrid HSE06 functional, the appropriate combination of Hartree–Fock (HF) mixing parameter, *α*, and the screening parameter, ω, were carefully considered to accurately reproduce the experimental band gap values of these oxides [50]. Unless stated otherwise, we used *α*  = 0.35 and ω= 0.05 Bohr^−1^ for MgO, and default values of *α* = 0.25 and ω= 0.11 Bohr^−1^ for the other metal oxides.

To gain further insight into the nature of the chemical bond on these oxides, we considered the oxygen vacancy energy formation, ${E}_{{O}_{{\text{vac}}}}$. In all cases ${E}_{{O}_{{\text{vac}}}}$ has been computed with VASP (PBE) as in Eq. 1 for all the metal oxide (M_x_O_y_) systems,

1 $${E}_{{O}_{{\text{vac}}}}= \left\{\left({{\text{E}}}_{{{\text{M}}}_{{\text{x}}}{{\text{O}}}_{{\text{y}}-1}}+{\frac{1}{2}E}_{{{\text{O}}}_{2}}\right)- {E}_{{{\text{M}}}_{{\text{x}}}{{\text{O}}}_{{\text{y}}}}\right\}$$

where, ${E}_{{M}_{x}{O}_{y}}$ is the energy of the corresponding metal oxide cell and ${E}_{{M}_{x}{O}_{y-1}}$ the same but with one O vacancy. The *x* and *y* are the number of metal (M) and oxygen (O) atoms in the chemical formula of metal oxides. The ${E}_{{O}_{2}}$ is the PBE energy of the O_2_ molecules in gas phase in its triplet ground state computed in a large box of 15 × 15 × 15 Å dimensions, here estimated to be − 10.766 eV.

## Results and discussion

**Fig. 1:**
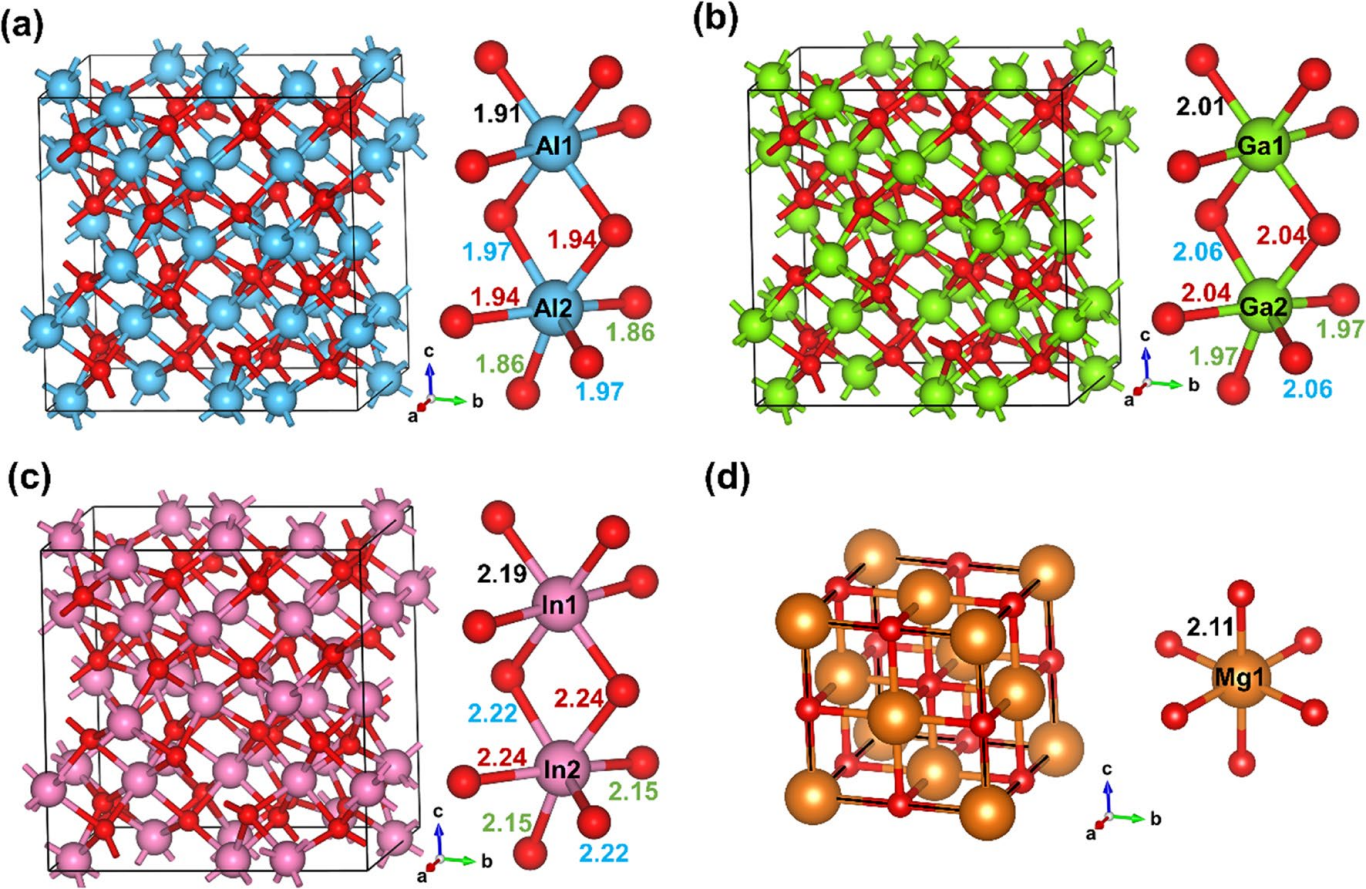
Crystal structures of (**a**)Al_2_O_3_, (**b**) Ga_2_O_3_, and (**c**) In_2_O_3_ containing 80 atoms, and (**d**)MgO with eight atoms. The oxygen coordination around the metal atoms given in the below panel. Blue, green, pink, orange, and red spheres represent the aluminum, gallium, indium, magnesium, and oxygen atoms, respectively. Different bond lengths are color-coded

We start the discussion focusing on the Group 13 Al_2_O_3_, Ga_2_O_3_, and In_2_O_3_ metal oxide models shown in Fig. 1, where MgO is included for comparison. The structural information of these metal oxides is extracted from the PBE-optimized bulk structures. Here, Al_2_O_3_, Ga_2_O_3_, and In_2_O_3_ have cubic structure with Ia̅3 space group. In this crystal structure, there are two types of inequivalent metal sites, hereafter denoted as M_1_ and M_2_. The M_1_ site is bonded to six equivalent O atoms, which forms a M_1_-O_6_ octahedron with aligned oxygen corners. The bond length between M_1_ and oxygens are 1.91, 2.01, and 2.19 Å for Al_2_O_3_, Ga_2_O_3_, and In_2_O_3_, respectively. On the other hand, the M_2_ site is bonded to six equivalent O atoms that form a M_2_-O_6_ octahedron but with distorted oxygen corners. Here, the bond length between M_2_ and oxygens are ranging from 1.86–1.97, 1.97–2.06, and 2.15–2.24 Å for Al_2_O_3_, Ga_2_O_3_, and In_2_O_3_, respectively. Therefore, M_1_ and M_2_ have different chemical environment which may affect their local electronic properties. For all these metal oxides, the oxygen atom is bonded to four metal atoms and generates trigonal pyramids with distorted metal atom corners. On other hand, MgO has a rock salt structure and cubic Fm̅3m space group. It is bonded to six equivalent O atoms to form a Mg-O_6_ octahedra structure with aligned oxygen corners (*cf.* Figure 1d). Similarly, oxygen is bonded to six equivalent Mg atoms with all Mg-O bond lengths are 2.11 Å. The lattice parameter of optimized structures is 8.91, 9.35, 10.23, and 4.19 Å for Al_2_O_3_, Ga_2_O_3_, In_2_O_3_, and MgO respectively, which are reported in Table 1. The computed lattice parameter of cubic In_2_O_3_ and MgO is in good agreement with the experimentally obtained lattice parameters of 10.12 [51] and 4.21 Å [52] and those of for cubic Al_2_O_3_, Ga_2_O_3_, In_2_O_3_ are also in agreement with previous computational studies [53, 54]. Note also that the PBE results obtained from VASP and FHI-AIMS are coincident as expected for the computational setup used and in agreement with the literature regarding the reproducibility of DFT-calculated values [55].

**Table 1:** The four studied metal oxides band gaps (*E*_*g*_, in eV) computed using different methods, their optimized lattice parameter (*a*, in Å), and the estimated vacancy energy formation (${E}_{{O}_{{\text{vac}}}}$, in eV). The three Group 13 oxides exhibit an indirect band gap. Experimental (Exp.) values are included for comparison

Oxides	*E*_*g*_					*a*	${E}_{{O}_{{\text{vac}}}}$
	VASP (PBE)	VASP (PBE + U)	FHI-AIMS (PBE)	FHI-AIMS (HSE06)	Exp.	VASP (PBE)	VASP (PBE)
MgO	4.7	4.7	4.7	7.7	7.8^58^	4.19	6.28
Al_2_O_3_	5.4	5.4	5.4	7.2	7.0–7.6^61^	8.91	6.11
Ga_2_O_3_	2.4	2.8	2.4	3.9	4.9–5.3^62^	9.35	4.60
In_2_O_3_	1.0	1.4	1.0	2.3	3.2^63^	10.23	2.75

Net charges on atoms have long been used to unveil the information about the chemical bonding in materials, even if these quantities are not physical observable and the values depend on the method used. Here, we rely on Bader charges [56] obtained from the numerical integration on atomic basins defined from a rigorous mathematical definition based on topological analysis of the electron density, which indeed is a physical observable. Nevertheless, one must advert that the thus obtained integrated charges often differ from what is expected from chemical intuition [57]. In the present work, we rely on trends rather than on the precise numerical values. With this caveat in mind, the results reported in Table 1 provide valuable information about the ionic character of the chemical bond in these materials. In the case of Al_2_O_3_, the same Bader charge value of + 3 is found for both M_1_ and M_2_ atoms. For Ga_2_O_3_ and In_2_O_3_, the Bader charges of M_1_ and M_2_ are slightly different, yet with deviations in the second or third decimal figure and, hence, not shown in Table 2. In the case of MgO, there is only one type of metal site and the Bader charge of Mg and O atoms are equal but with opposite signs. From the Bader charge analysis, it is seen that the ionicity of the Group 13 metal oxides decreases from Al_2_O_3_ to In_2_O_3_. Nevertheless, the charge separation is large implying that the chemical bonding in these metal oxides is largely ionic in nature. Based on Bader charges and the formal oxidation states, it is possible to make a rough estimate of the contribution of ionic bond in each compound. MgO is introduced here as a prototype of fully ionic oxide, as evidenced from the analysis of HF and configuration interaction cluster model wavefunctions. Nevertheless, the DFT picture arising from the periodic calculations with either PBE and PBE + U is slightly different as Bader charges are of 85% of the formal oxidation state. The deviation from a fully ionic picture can be safely attributed to the tendency of DFT to delocalize the electron density, as these functionals are derived from the electron gas uniform density. The strong ionic character of MgO is consistent with a large band gap of 7.7 eV as predicted by the HSE06 functional (*cf.* Table 1) which nicely agrees with the experimental value of 7.8 eV [58]. Let us now focus on the trend of ionicity in Group 13 oxides. The results for cubic Al_2_O_3_ studied here indicate that the bonding is fully ionic. This is in complete agreement with previous HF cluster model results for the slightly more stable trigonal (R $\stackrel{\mathrm{-}}{3}$ c) polymorph [26, 27]. However, the situation is significantly different when going to Ga_2_O_3_ and In_2_O_3_ where the Bader charge at the metal and O atoms is around + 1.9 *e* and − 1.2 *e*, which, compared to the formal oxidation state, implies roughly a 60% of ionicity only. This is consistent with much smaller band gaps and has implications for doping of these oxides as the dopant will naturally to exhibit a + 2 oxidation state.

**Table 2:** The four studied metal oxides with oxidation state (*#*) on metal atoms and Bader charges (*Q*, in *e*) on metal and oxygen atoms computed using PBE and PBE + U method

Oxides		*Q* (PBE)			*Q* (PBE + U)		
	#	M_1_	M_2_	O	M_1_	M_2_	O
MgO	+ 2	+ 1.7	—	− 1.7	+ 1.7	—	− 1.7
Al_2_O_3_	+ 3	+ 3.0	+ 3.0	− 2.0	+ 3.0	+ 3.0	− 2.0
Ga_2_O_3_	+ 3	+ 1.9	+ 1.9	− 1.2	+ 1.8	+ 1.9	− 1.2
In_2_O_3_	+ 3	+ 1.9	+ 1.9	− 1.3	+ 1.9	+ 1.9	− 1.2

To better understand the chemistry of these metal oxides and to model catalysts based in these materials, it is essential to determine the band gap, *E*_*g*_, as well as the alignment or orientation of occupied and unoccupied levels accurately enough, here taking systematically the valence band maximum as the Fermi energy level, *E*_*F*_. Figure S2 of the SI displays the PBE + U band structure of the Al_2_O_3_, Ga_2_O_3_, and In_2_O_3_ as obtained from VASP which shows a clear indirect band gap, it also includes that of MgO presenting a direct band gap at $\Gamma$ point. The values of band gap obtained from the different functionals are given in Table 1. The positions of the valence band maximum and the conduction band minimum play a pivotal role in delineating the redox characteristics of the catalyst, given their significance as electronic states engaged in the transfer of charge to or from the catalyst. As already mentioned, there is a tendency for band gaps to decrease with decreasing ionicity owing to increase in the dispersion of the band in the reciprocal space produced by a larger overlap of the metal and oxygen orbitals. Similarly, the density of states (DOS) analysis is consistence with the band structure plots (*cf.* Figure S3), implying that band structure plots are already sampling the key characteristic points of the electronic structure. Here, we plotted the projected DOS of each element to understand the individual contribution of metal and oxygen near *E*_*F*_. It is found that the occupied states near *E*_*F*_ (states < 0 eV) correspond to the O 2*p* orbitals whereas the higher energy conduction band is dominated by the metal valence orbitals. In these metal oxides, the valence band extends approximately 5 to 8 eV and primarily consists of O 2*p* states with significant hybridization with the metal *ns* and *np* orbitals (*n* = 3, 4, 5 for Al, Ga, In, respectively). Moreover, certain *d* states play a role in shaping the upper valence band structure, thus impacting the bonding characteristics of the material. Here, it is seen that the less metallic states occur near *E*_*F*_ and lead to strong ionic bonding in Al_2_O_3_ and MgO, in agreement with the picture derived from the Bader charges. On the other hand, more metallic states appear near the Fermi level in Ga_2_O_3_ and In_2_O_3_ implying a lesser ionic contribution than in Al_2_O_3_ and MgO, again in agreement with the Bader charge analysis.

**Fig. 2:**
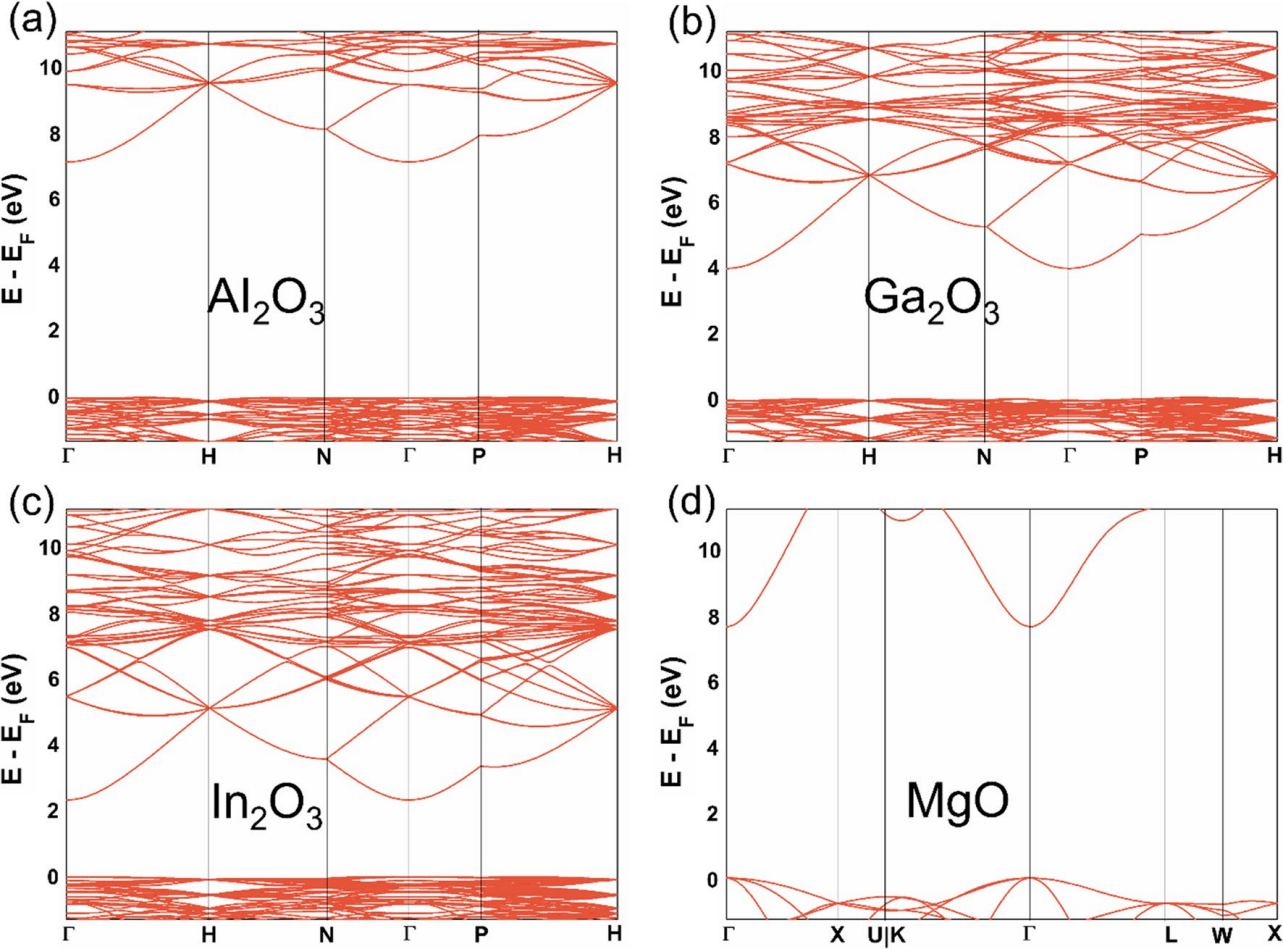
Band structure of the studied oxides computed with FHI-AIMS using the HSE06 method for **a** Al_2_O_3_, **b** Ga_2_O_3_, **c** In_2_O_3_, and **d** MgO. The default α and ω values are used for Group 13 metal oxides. The *E*_*F*_ is set at zero energy

**Fig. 3:**
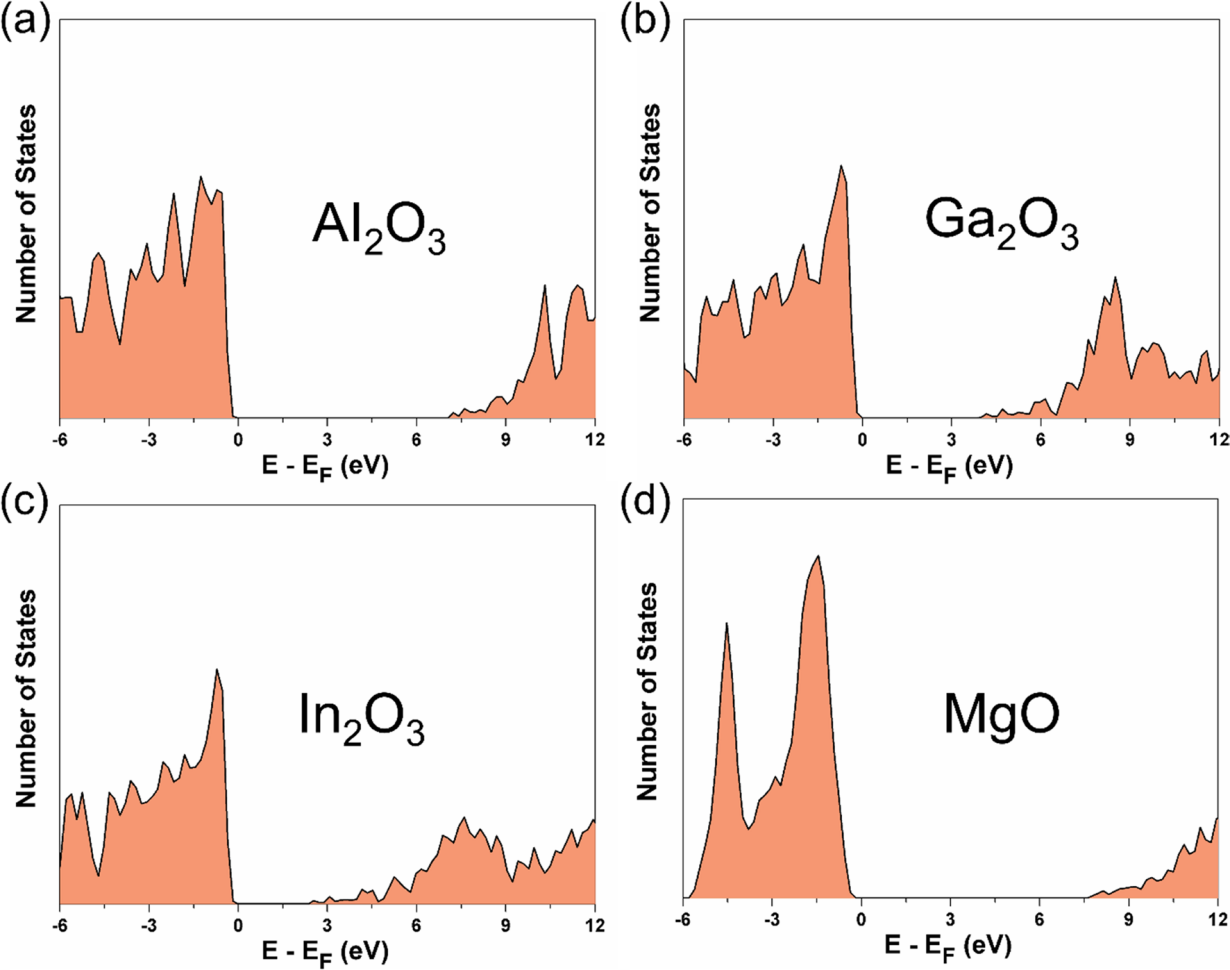
Total DOS per unit volume (Å^3^) computed with FHI-AIMS using the HSE06 method for **a** Al_2_O_3_, **b** Ga_2_O_3_, **c** In_2_O_3_, **d** MgO. The default *α* and ω values are used for Group 13 metal oxides. The *E*_*F*_ is set at zero energy

There are studies which already reported the relationship of band gap with lattice constant in metal oxides [59, 60]. The lattice constant denotes the side length of the unit cell in a crystal lattice. The lattice itself signifies the spatial separation between constituent molecules or atoms forming the lattice structure. In general, a reduction in lattice constant implies that electrons are bound more closely to the respective atoms, necessitating a higher energy input for their removal. Consequently, this leads to the expanded band gap. Conversely, as a consequence of the diminishing lattice parameter, both the valence (occupied) and the conduction (unoccupied) bandwidths will also decrease. Similarly, we also obtained the inverse linear relationship between band gap and lattice constant for Al_2_O_3_, Ga_2_O_3_, and In_2_O_3_, a trend that does not depend on the functional used even if the band gap values exhibit a large dependency of DFT method chosen. At this point it is worth pointing out that the band gap values and DOS are underestimated when employing the VASP with GGA as well as GGA + U method (*cf.* Table 1), as expected. To get the deep insight into electronic properties of these metal oxides, we performed FHI-AIMS calculations with PBE and HSE06 functional at the VASP (PBE) structure. As in the case of lattice parameter, PBE values for the band gap calculated with VASP or FHI-AIMS are virtually identical. The PBE + U band gap values are close to the PBE in VASP for MgO and Al_2_O_3_, probably due to their strong ionic character, and difference is slightly larger for Ga_2_O_3_ and In_2_O_3_, as expected from the lower ionicity and increased dispersion of the occupied bands. On the other hand, the HSE06 band gaps for Al_2_O_3_, Ga_2_O_3_, and In_2_O_3_ in Table 1 are close but quite lower to the experimentally reported band gap values [61–63]. Note, however, that the experimental values for Al_2_O_3_ and Ga_2_O_3_ correspond to the range for several polymorphs. Figures 2 and 3 report the HSE06 band structure and DOS of the studied oxides whereas the corresponding pictures for VASP (PBE), VASP (PBE + U), and FHI-AIMS (PBE) are given in the supporting information (Figures S2, S4, and S5). In Figure S6 of the SI, we provide a comparison between DOS computed through VASP (PBE), VASP (PBE + U), FHI-AIMS (PBE), and FHI-AIMS (HSE06). It is observed that the VASP estimates higher density of states computed relative to FHI-AIMS due to the different integration methods, while the same shape is gained, plus the FHI-AIMS DOS with PBE and HSE06 functionals exhibit a similar trend. Finally, to gain information about the influence of Hartree–Fock mixing parameter, *α*, and screening parameter, ω, in HSE06 calculations on band gap of these metal oxides we carried out a series of additional calculations with the FHI-AIMS code. In detail, we considered four cases, where both parameters are slightly modified, as follows; case I (default values): *α* = 0.25 and ω= 0.11 Bohr^−1^, case II: *α* = 0.25 and ω = 0.05 Bohr^−1^, case III: *α* = 0.3 and ω  = 0.05 Bohr^−1^, case IV: *α* = 0.35 and ω  = 0.05 Bohr^−1^. It is observed that, increasing *α* and reducing ω, respectively, the calculated band gap values increase, eventually matching the value corresponding to the experimental band gap (*cf.* Table 3), in line with previous work for TiO_2_ polymorphs [50]. However, the overall trend of band gap values and correlation with other properties remain unchanged.

**Table 3:** The four studied metal oxides with four cases of *α* and ω values (ω, in Bohr^−1^) in FHI-AIMS (HSE06) calculations and their computed band gap (*E*_*g*_ in eV) values. Note that *α* = 0.25 and ω = 0.11 Bohr^−1^ are the default values of HSE06 functional

	*E*_*g*_			
Oxides	*α* = 0.25 ω = 0.11	*α* = 0.25 ω = 0.05	*α* = 0.3 ω = 0.05	*α* = 0.35 ω = 0.05
MgO	6.4	6.8	7.2	7.7
Al_2_O_3_	7.2	7.6	8.0	8.4
Ga_2_O_3_	3.9	4.3	4.7	5.1
In_2_O_3_	2.3	2.7	3.0	3.4

To end up this part, we focus on the cost of the oxygen vacancies formation that ultimately dictate the catalytic efficacy of metal oxides and that is also related to the nature of the chemical bond. These morphological defects have a favorable impact on reactions by reducing barriers or stabilizing crucial intermediates [64, 65]. The formation energy of oxygen vacancies in compounds typically depends on the atomic chemical potentials or environmental conditions within the system. Therefore, we calculated the formation energy of oxygen vacancy in these four metal oxides as in Eq. 1 and included the values in Table 1. Here, the lower formation energy makes faster oxygen ejection from the metal oxides, thus increasing the reducibility. Among these four metal oxides, In_2_O_3_ appears to have the smallest ${E}_{{O}_{{\text{vac}}}}$ found and MgO the highest. Note also that in the case of In_2_O_3_, the O vacancy acts as a shallow donor due to its low formation energy and its tendency to transition directly to lower charge states at the calculated conduction band maximum. Moreover, it can serve as the predominant donor defect, elucidating both the *n*-type conductivity and the non-stoichiometry [16]. It has been reported that the ${E}_{{O}_{{\text{vac}}}}$ in the metal oxides are correlated with their band gap values [66]. Here, we also found the same linear trend between ${E}_{{O}_{{\text{vac}}}}$, band gap and lattice parameters (*cf.* Figure S7).

## Conclusions

The nature of the chemical bonding in cubic Group 13 metal oxides, with MgO added as an ionic reference system, has been studied using a variety of DFT methods and functionals of increasing accuracy and their electronic structure unveiled. Our refined crystal structure models align closely with the experimental findings as reported. Analysis of Bader charges confirms that MgO and Al_2_O_3_ exhibit almost full ionic character, while Ga_2_O_3_ and In_2_O_3_ display only approximately 60% ionicity. These findings hold implications for doping, as dopants within Al_2_O_3_ and MgO will tend to adopt a + 3 oxidation state, while doping Ga_2_O_3_ and In_2_O_3_ will result in dopants exhibiting a + 2 oxidation state.

Furthermore, we saw that the band gap of these metal oxides decreases with decreasing ionicity as expected. Also, the study of oxygen vacancy formation shows that the energy cost to form this point defect reduces with band gap as well as ionicity. Therefore, the lowest ${E}_{{O}_{{\text{vac}}}}$ is obtained for the In_2_O_3_.

Among the scrutinized DFT methodologies, the band gaps computed using the hybrid HSE06 functional in FHI-AIMS calculations closely match experimental values. Nevertheless, for this hybrid functional one must be aware that there is an impact of the chosen values of the screening and mixing parameters on the band gap of metal oxides. Overall, that increasing the screening parameter, *α*, and decreasing the screening parameter, ω, lead to larger band gap values, eventually matching the experimental value although this adds a semiempirical flavor to this approach. Interestingly, the shift produced by varying these parameters is almost rigid as affects the three oxides in the same way. The origin of this behavior requires perhaps a more detailed analysis which is out of the scope of the present work.

Our thorough investigation into the chemical bonding and electronic properties of Group 13 metal oxides can help to design appropriate surface model and thus accelerate the discovery of descriptors that correlate these physical properties with catalyst activity. This aspect is crucial in advancing the development of metal oxides as highly efficient catalysts and catalytic supports.

### Supplementary Information

Below is the link to the electronic supplementary material.

Supplementary file1 (DOCX 2233 KB)

## Data Availability

All input files are available upon request to the authors.

## References

[1] Fierro JLG (2005). Metal oxides: chemistry and applications.

[2] Trovarelli A (2002). Catalysis by ceria and related materials.

[3] Peña MA, Fierro JLG (2001). Chemical structures and performance of perovskite oxides. Chem Rev.

[4] Khdary NH, Alayyar AS, Alsarhan LM, Alshihri S, Mokhtar M (2022). Metal oxides as catalyst/supporter for CO_2_ capture and conversion. Review Catalysts.

[5] Ozorio LP, Mota CJA (2017). Direct carbonation of glycerol with CO_2_ catalyzed by metal oxides. ChemPhysChem.

[6] Pawelec B (2005) Surface processes and composition of metal oxide surfaces. In: Metal oxides. CRC Press

[7] Védrine JC (2017). Heterogeneous catalysis on metal oxides Catalysts.

[8] Zhang M, Jeerh G, Zou P, Lan R, Wang M, Wang H, Tao S (2021). Recent development of perovskite oxide-based electrocatalysts and their applications in low to intermediate temperature electrochemical devices. Mater Today.

[9] Guo T, Yao M-S, Lin Y-H, Nan C-W (2015). A comprehensive review on synthesis methods for transition-metal oxide nanostructures. CrystEngComm.

[10] Mabate TP, Maqunga NP, Ntshibongo S, Maumela M, Bingwa N (2023). Metal oxides and their roles in heterogeneous catalysis: special emphasis on synthesis protocols, intrinsic properties, and their influence in transfer hydrogenation reactions. SN Appl Sci.

[11] Pirkanniemi K, Sillanpää M (2002). Heterogeneous water phase catalysis as an environmental application: a review. Chemosphere.

[12] Li Y, Zhang Y, Qian K, Huang W (2022). Metal–support interactions in metal/oxide catalysts and oxide–metal interactions in oxide/metal inverse catalysts. ACS Catal.

[13] Ren Y, Xie W, Li Y, Ma J, Li J, Liu Y, Zou Y, Deng Y (2021). Noble metal nanoparticles decorated metal oxide semiconducting nanowire arrays interwoven into 3D mesoporous superstructures for low-temperature gas sensing. ACS Cent Sci.

[14] Ma J, Xiao X, Zou Y, Ren Y, Zhou X, Yang X, Cheng X, Deng Y (2019). A general and straightforward route to noble metal-decorated mesoporous transition-metal oxides with enhanced gas sensing performance. Small.

[15] Hicks RF, Qi H, Young ML, Lee RG (1990). Structure sensitivity of methane oxidation over platinum and palladium. J Catal.

[16] Agoston P, Erhart P, Klein A, Albe K (2009). Geometry, electronic structure and thermodynamic stability of intrinsic point defects in indium oxide. J Phys Condens Matter.

[17] Ruhaimi AH, Aziz MAA, Jalil AA (2021) Magnesium oxide-based adsorbents for carbon dioxide capture: current progress and future opportunities. J CO_2_ Util 43:101357

[18] Dziejarski B, Serafin J, Andersson K, Krzyżyńska R (2023). CO_2_ capture materials: a review of current trends and future challenges. Mater Today Sustain.

[19] Trueba M, Trasatti SP (2005). γ-Alumina as a support for catalysts: a review of fundamental aspects. Eur J Inorg Chem.

[20] Wang C-H (2004). Al_2_O_3_-supported transition-metal oxide catalysts for catalytic incineration of toluene. Chemosphere.

[21] Wang J, Zhang G, Zhu J, Zhang X, Ding F, Guo X, Song C (2021). CO_2_ hydrogenation to methanol over In_2_O_3_-based catalysts: from mechanism to catalyst development. ACS Catal.

[22] Cai D, Cai Y, Tan KB, Zhan G (2023). Recent advances of indium oxide-based catalysts for CO_2_ hydrogenation to methanol: experimental and theoretical. Materials.

[23] Shao C-T, Lang W-Z, Yan X, Guo Y-J (2017). Catalytic performance of gallium oxide based-catalysts for the propane dehydrogenation reaction: effects of support and loading amount. RSC Adv.

[24] Davies T, Taylor SH (2004). The oxidative dehydrogenation of propane using gallium–molybdenum oxide-based catalysts. J Mol Catal A: Chem.

[25] Illas F, Lorda A, Rubio J, Torrance JB, Bagus PS (1993). The nature of the chemical bond in simple oxides: a theoretical journey from the ionic model to the *ab initio* configuration interaction approach. J Chem Phys.

[26] Pacchioni G, Sousa C, Illas F, Parmigiani F, Bagus PS (1993). Measures of ionicity of alkaline-earth oxides from the analysis of ab initio cluster wave functions. Phys Rev B.

[27] Sousa C, Illas F, Pacchioni G (1993). Can corundum be described as an ionic oxide?. J Chem Phys.

[28] Sousa C, Illas F (1994). Ionic-covalent transition in titanium oxides. Phys Rev B.

[29] Gurlo A (2010). Structural stability of high-pressure polymorphs in In_2_O_3_ nanocrystals: evidence of stress-induced transition?. Angew Chem Int Ed.

[30] Zhao J, Byggmästar J, He H, Nordlund K, Djurabekova F, Hua M (2023). Complex Ga_2_O_3_ polymorphs explored by accurate and general-purpose machine-learning interatomic potentials. npj Comput Mater.

[31] McCarthy MI, Harrison NM (1994). Ab initio determination of the bulk properties of MgO. Phys Rev B.

[32] Jain A, Ong SP, Hautier G, Chen W, Richards WD, Dacek S, Cholia S, Gunter D, Skinner D, Ceder G, Persson KA (2013). The materials project: a materials genome approach to accelerating materials innovation. APL Mater.

[33] Kresse G, Furthmüller J (1996). Efficient iterative schemes for ab initio total-energy calculations using a plane-wave basis set. Phys Rev B.

[34] Perdew JP, Burke K, Ernzerhof M (1996). Generalized gradient approximation made simple. Phys Rev Lett.

[35] Kresse G, Joubert D (1999). From ultrasoft pseudopotentials to the projector augmented-wave method. Phys Rev B.

[36] Blöchl PE (1994). Projector augmented-wave method. Phys Rev B.

[37] Monkhorst HJ, Pack JD (1976). Special points for Brillouin-zone integrations. Phys Rev B.

[38] Erhart P, Klein A, Egdell RG, Albe K (2007). Band structure of indium oxide: indirect versus direct band gap. Phys Rev B.

[39] Muscat J, Wander A, Harrison NM (2001). On the prediction of band gaps from hybrid functional theory. Chem Phys Lett.

[40] Moreira IPR, Illas F, Martin RL (2002). Effect of Fock exchange on the electronic structure and magnetic coupling in NiO. Phys Rev B.

[41] Adamo C, Barone V (1999). Toward reliable density functional methods without adjustable parameters: the PBE0 model. J Chem Phys.

[42] Heyd J, Scuseria GE, Ernzerhof M (2003). Hybrid functionals based on a screened Coulomb potential. J Chem Phys.

[43] Viñes F, Illas F (2017). Electronic structure of stoichiometric and reduced ZnO from periodic relativistic all electron hybrid density functional calculations using numeric atom-centered orbitals. J Comput Chem.

[44] Ko KC, Lamiel-García O, Lee JY, Illas F (2016). Performance of a modified hybrid functional in the simultaneous description of stoichiometric and reduced TiO_2_ polymorphs. Phys Chem Chem Phys.

[45] Das T, Di Liberto G, Tosoni S, Pacchioni G (2019). Band gap of 3D metal oxides and quasi-2D materials from hybrid density functional theory: are dielectric-dependent functionals superior?. J Chem Theory Comput.

[46] Dudarev SL, Botton GA, Savrasov SY, Humphreys CJ, Sutton AP (1998). Electron-energy-loss spectra and the structural stability of nickel oxide: an LSDA+U study. Phys Rev B.

[47] Nolan M, Watson GW (2005). The electronic structure of alkali doped alkaline earth metal oxides: Li doping of MgO studied with DFT-GGA and GGA+U. Surf Sci.

[48] Blum V, Gehrke R, Hanke F, Havu P, Havu V, Ren X, Reuter K, Scheffler M (2009). Ab initio molecular simulations with numeric atom-centered orbitals. Comput Phys Commun.

[49] Ren X, Rinke P, Blum V, Wieferink J, Tkatchenko A, Sanfilippo A, Reuter K, Scheffler M (2012). Resolution-of-identity approach to Hartree-Fock, hybrid density functionals, RPA, MP2 and GW with numeric atom-centered orbital basis functions. New J Phys.

[50] Viñes F, Lamiel-García O, Ko KC, Lee JY, Illas F (2017). Systematic study of the effect of HSE functional internal parameters on the electronic structure and band gap of a representative set of metal oxides. J Comput Chem.

[51] Marezio M (1966). Refinement of the crystal structure of In_2_O_3_ at two wavelengths. Acta Crystallogr.

[52] Wyckoff R (1963). Crystal structures.

[53] Sabino FP, Nunes de Oliveira L, Da Silva JLF (2014). Role of atomic radius and d-states hybridization in the stability of the crystal structure of M_2_O_3_ (M =Al, Ga, In) oxides. Phys Rev B.

[54] Ramzan M, Li Y, Ahuja R (2013). Electronic structure, mechanical and optical properties of In_2_O_3_ with hybrid density functional (HSE06). Solid State Commun.

[55] Lejaeghere K, Bihlmayer G, Björkman T, Blaha P, Blügel S, Blum V, Caliste D, Castelli IE, Clark SJ, Dal Corso A, De Gironcoli S, Deutsch T, Dewhurst JK, Di Marco I, Draxl C, Dułak M, Eriksson O, Flores-Livas JA, Garrity KF, Genovese L, Giannozzi P, Giantomassi M, Goedecker S, Gonze X, Grånäs O, Gross EKU, Guland A, Gygi F, Hamann DR, Hasnip PJ, Holzwarth NAW, Iuşan D, Jochym DB, Jollet F, Jones D, Kresse G, Koepernik K, Küçükbenli E, Kvashnin YO, Inka L, Locht M, Lubeck S, Marsman M, Marzari N, Nitzsche U, Nordström L, Taisuke O, Paulatto L, Pickard CJ, Poelmans W, Probert MIJ, Refson K, Richter M, Rignanese G, Saha S, Scheffler M, Schlipf M, Schwarz K, Sharma S, Tavazza F, Thunström P, Tkatchenzo, Torrent M, Vanderbilt D, Van Setten MJ, Van Speybroeck V, Wills JM, Yates JR, Zhang GX, Cottenier S (2016). Reproducibility in density functional theory calculations of solids. Science.

[56] Bader RFW (1991). A quantum theory of molecular structure and its applications. Chem Rev.

[57] Politzer P, Murray JS (2019). A look at bonds and bonding. Struc Chem.

[58] Roessler DM, Walker WC (1967). Electronic spectrum and ultraviolet optical properties of crystalline MgO. Phys Rev.

[59] Shi Y, Lian J, Hu W, Liu Y, He G, Jin K, Song H, Dai K, Fang J (2019). Study the relation between band gap value and lattice constant of MgTi_2_O_4_. J Alloys Compd.

[60] Wang T, Moll N, Cho K, Joannopoulos JD (1999). Deliberately designed materials for optoelectronics applications. Phys Rev Lett.

[61] Filatova EO, Konashuk AS (2022). Interpretation of the changing the band gap of Al_2_O_3_ Depending on Its Crystalline Form: Connection with Different Local Symmetries. J Phys Chem C.

[62] Biswas M, Nishinaka H (2022). Thermodynamically metastable α-, ε- (or κ-), and γ-Ga_2_O_3_: from material growth to device applications. APL Mater.

[63] de Boer T, Bekheet MF, Gurlo A, Riedel R, Moewes A (2016). Band gap and electronic structure of cubic, rhombohedral, and orthorhombic In_2_O_3_ polymorphs: experiment and theory. Phys Rev B.

[64] Pacchioni G (2003). Oxygen vacancy: the invisible agent on oxide surfaces. ChemPhysChem.

[65] Zong X, Jin Y, Li Y, Zhang X, Zhang S, Xie H, Zhang J, Xiong Y (2022) Morphology-controllable ZnO catalysts enriched with oxygen-vacancies for boosting CO_2_ electroreduction to CO. J CO_2_ Util 61:102051

[66] Deml AM, Stevanović V, Muhich CL, Musgrave CB, O’Hayre R (2014). Oxide enthalpy of formation and band gap energy as accurate descriptors of oxygen vacancy formation energetics. Energy Environ Sci.

