# Supplementary material for: Chemical bonding and electronic properties along Group 13 metal oxides
Source: J Mol Model. 2024 May 7;30(6):161. doi: 10.1007/s00894-024-05957-6 (PMC11076323; doi:10.1007/s00894-024-05957-6)

**Chemical Bonding and Electronic Properties along the Group 13 Metal Oxides**

Samadhan Kapse, Maria Voccia, Francesc Viñes, and Francesc Illas^*^

*Departament de Ciència de Materials i Química Física & Institut de Química Teòrica i Computacional (IQTCUB), Universitat de Barcelona,
c/Martí i Franquès 1-11, 08028 Barcelona, Spain*

*corresponding author, e-mail: [francesc.illas@ub.edu](mailto:francesc.illas@ub.edu)

**CONTENT**

**Figure S1**. Schematic of **k**-path along the reducible Brillouin zone

**Figure S2**. Bandstructures computed using VASP (PBE+U) method

**Figure S3**. Projected DOS computed using VASP (PBE+U) method

**Figure S4**. Bandstructures computed using VASP (PBE) method

**Figure S5**. Bandstructures computed using FHI-AIMS (PBE) method

**Figure S6**. Comparison between DOS computed through VASP (PBE), VASP (PBE+U), FHI-AIMS (PBE), and FHI-AIMS (HSE06) methods

**Figure S7**. Correlation between $E_{O_{vac}}$ , band gap and lattice parameters

**Figure S1.** Schematic of **k**-path along the reducible Brillouin zone for (a) MgO and (b) for the other metal oxides (Al_2_O_3_, In_2_O_3_, and Ga_2_O_3_).

**
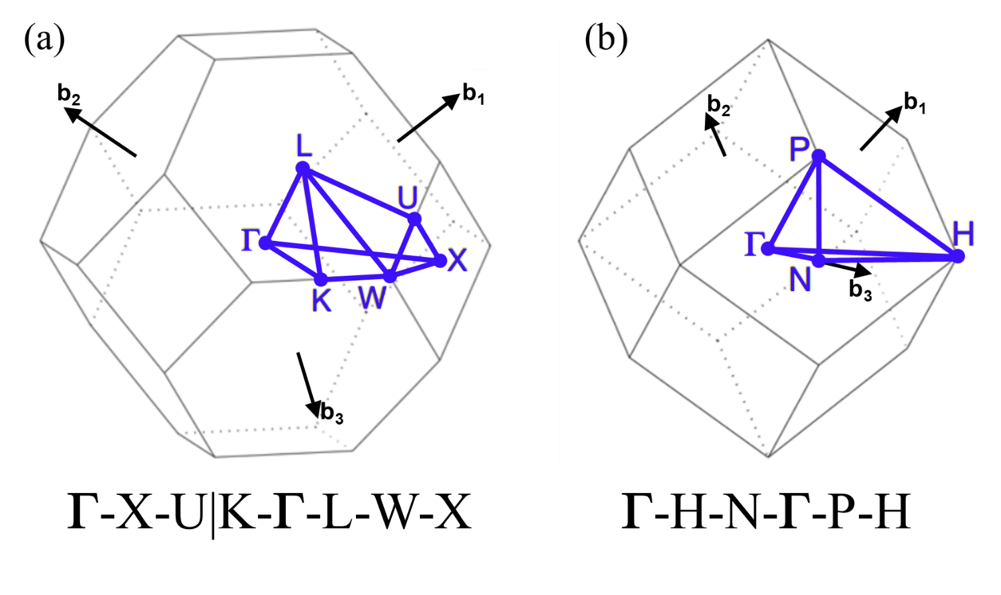
**

**Figure S2.** Bandstructures computed using VASP (PBE+U) method for (a) Al_2_O_3_, (b) Ga_2_O_3_, (c) In_2_O_3_, and (d) MgO. The Fermi level is set at zero energy.

**
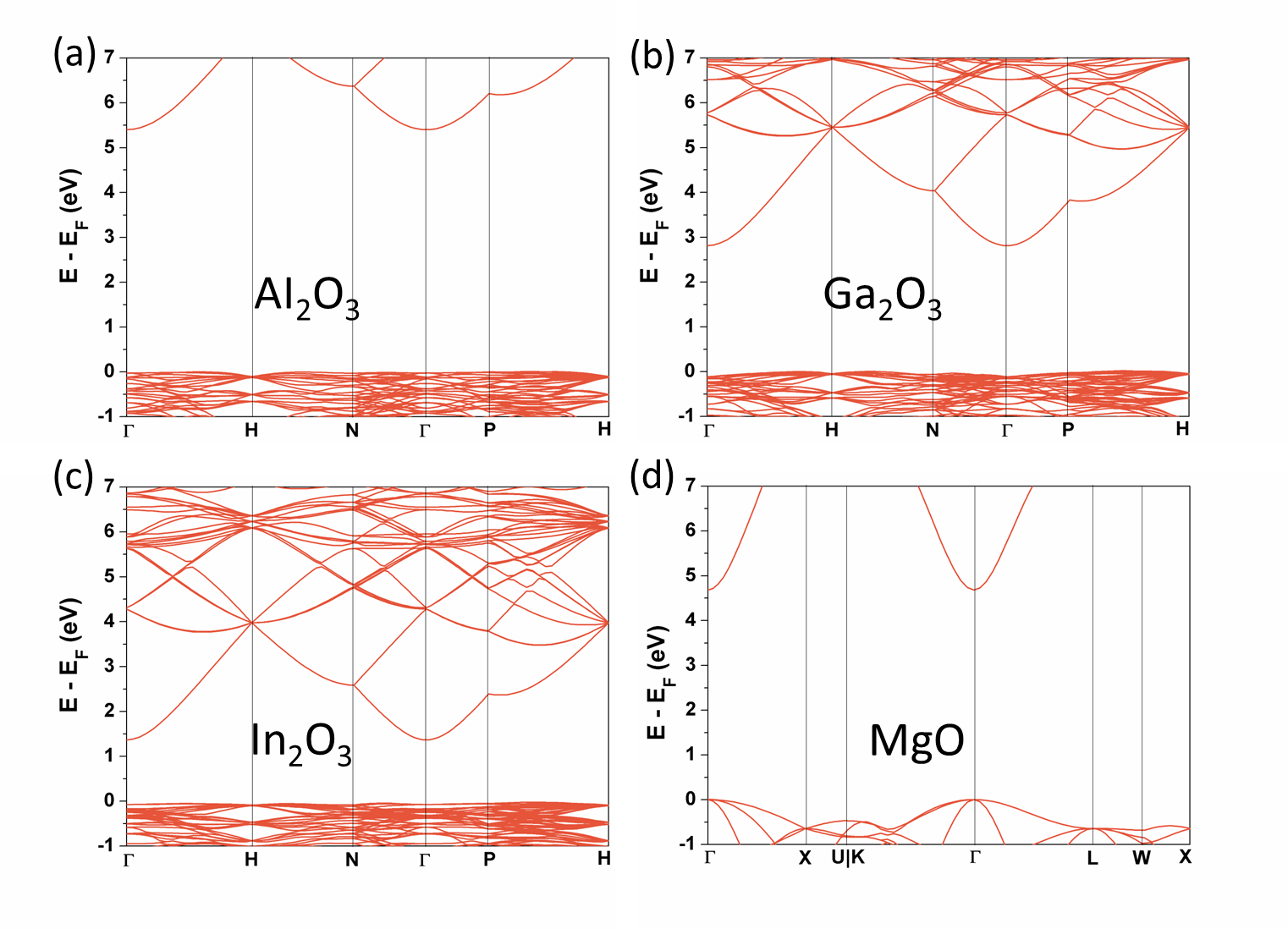
**

**Figure S3.** Projected DOS (PDOS) per unit volume (Å^3^) computed using VASP (PBE+U) method for (a) Al_2_O_3_, (b) Ga_2_O_3_, (c) In_2_O_3_, (d) MgO. The *E*_F_ is set at zero energy.

**
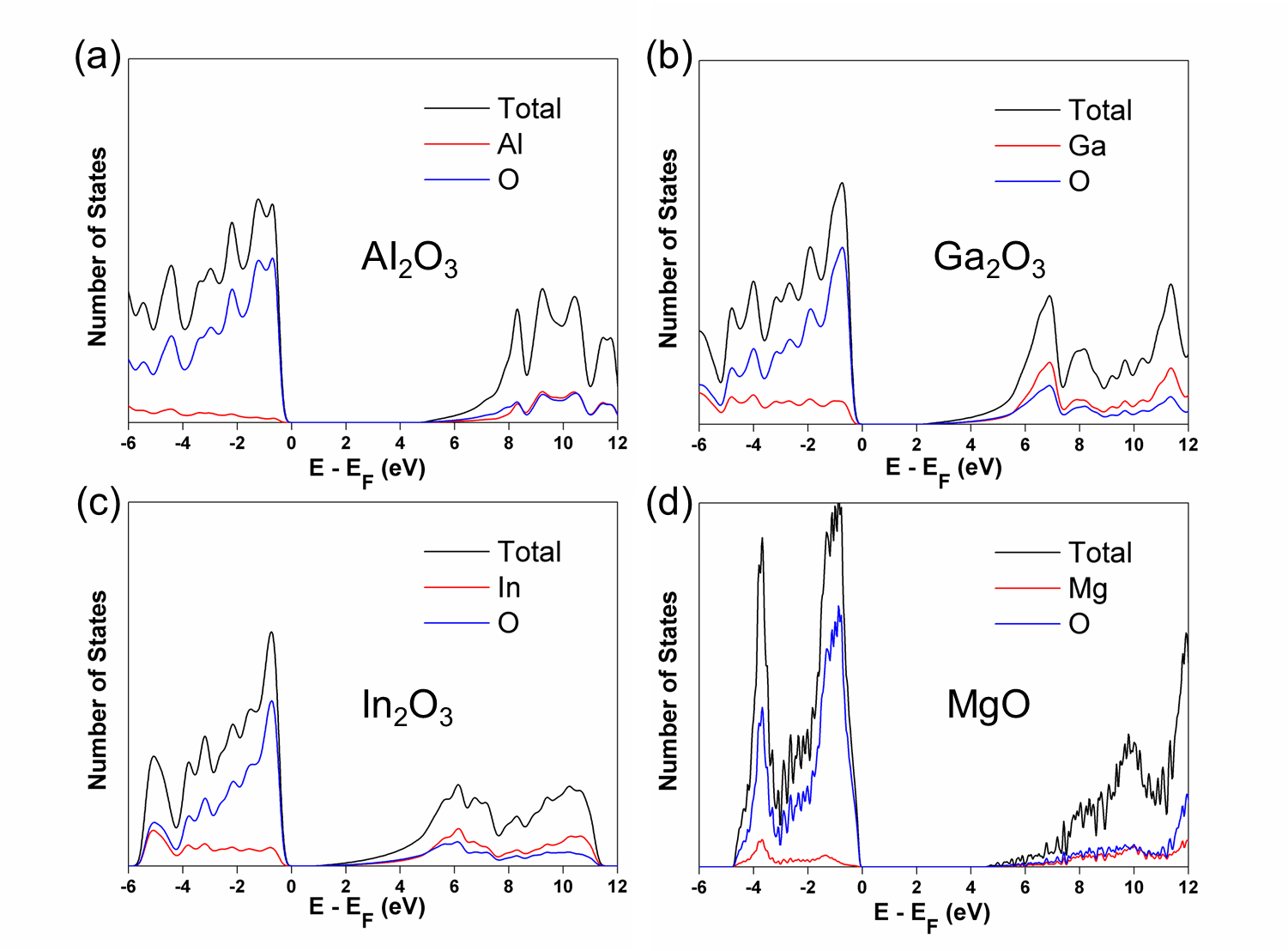
**

**Figure S4.** Bandstructures computed using VASP (PBE) method for (a) Al_2_O_3_, (b) Ga_2_O_3_, (c) In_2_O_3_, and (d) MgO. The *E*_F_ is set at zero energy.


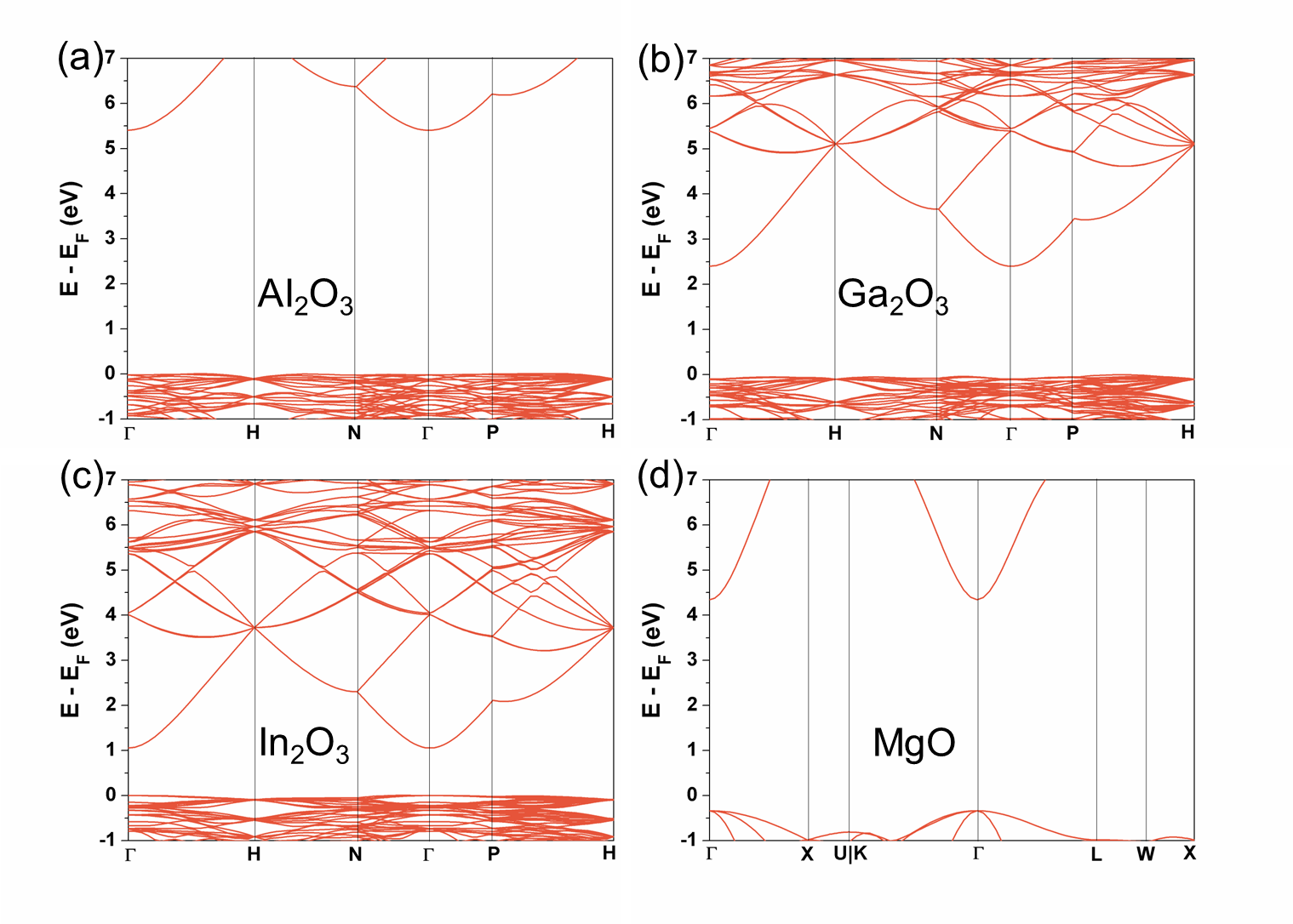


**Figure S5.** Bandstructures computed using FHI-AIMS (PBE) method for (a) Al_2_O_3_, (b) Ga_2_O_3_, (c) In_2_O_3_, and (d) MgO. The *E*_F_ is set at zero energy.

**
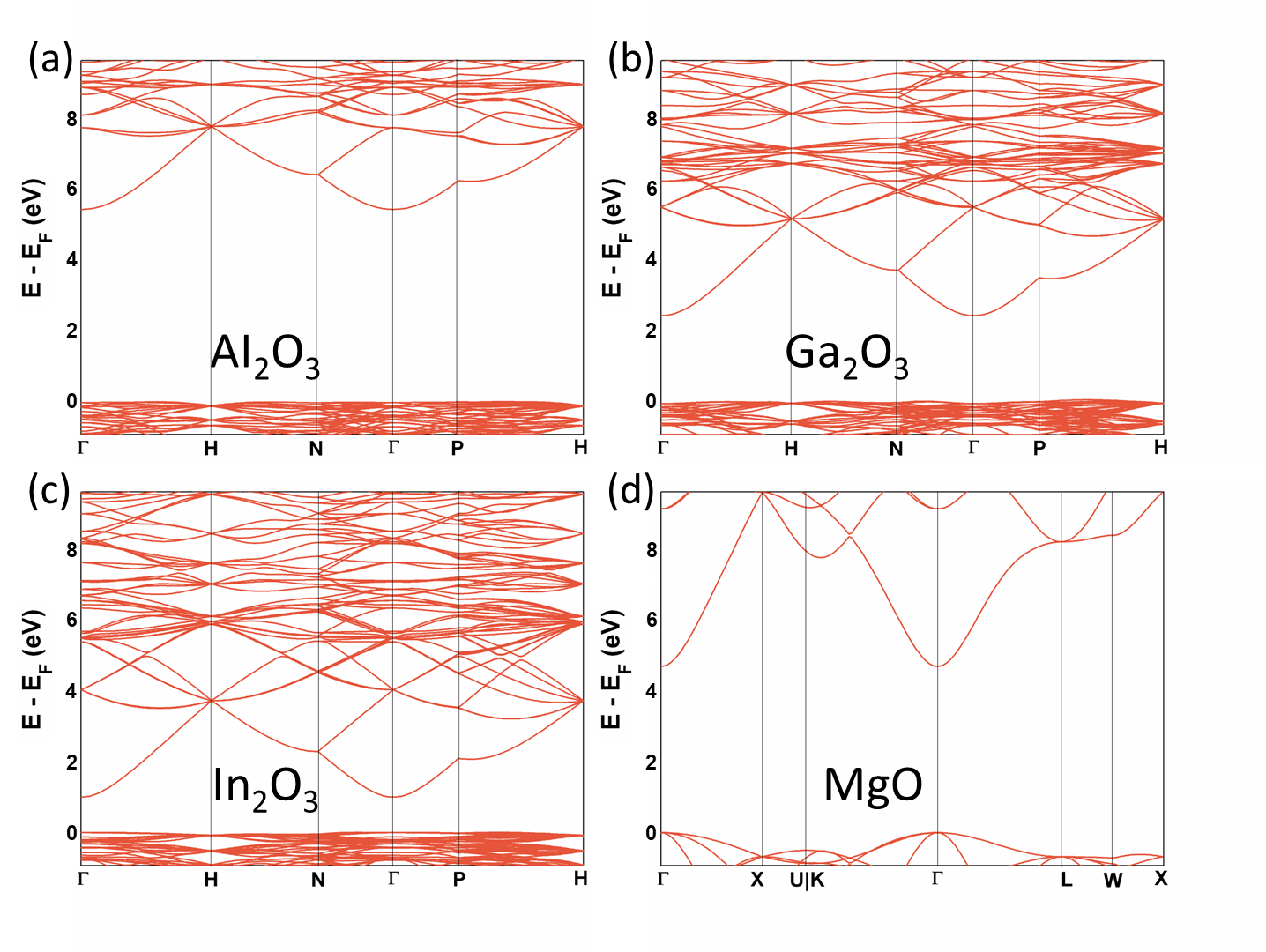
**

**Figure S6.** Comparison between total DOS computed through VASP (PBE), VASP (PBE+U), FHI-AIMS (PBE), and FHI-AIMS (HSE06) methods for (a) Al_2_O_3_, (b) Ga_2_O_3_, (c) In_2_O_3_, and (d) MgO. The *E*_F_ is set at zero energy.

**
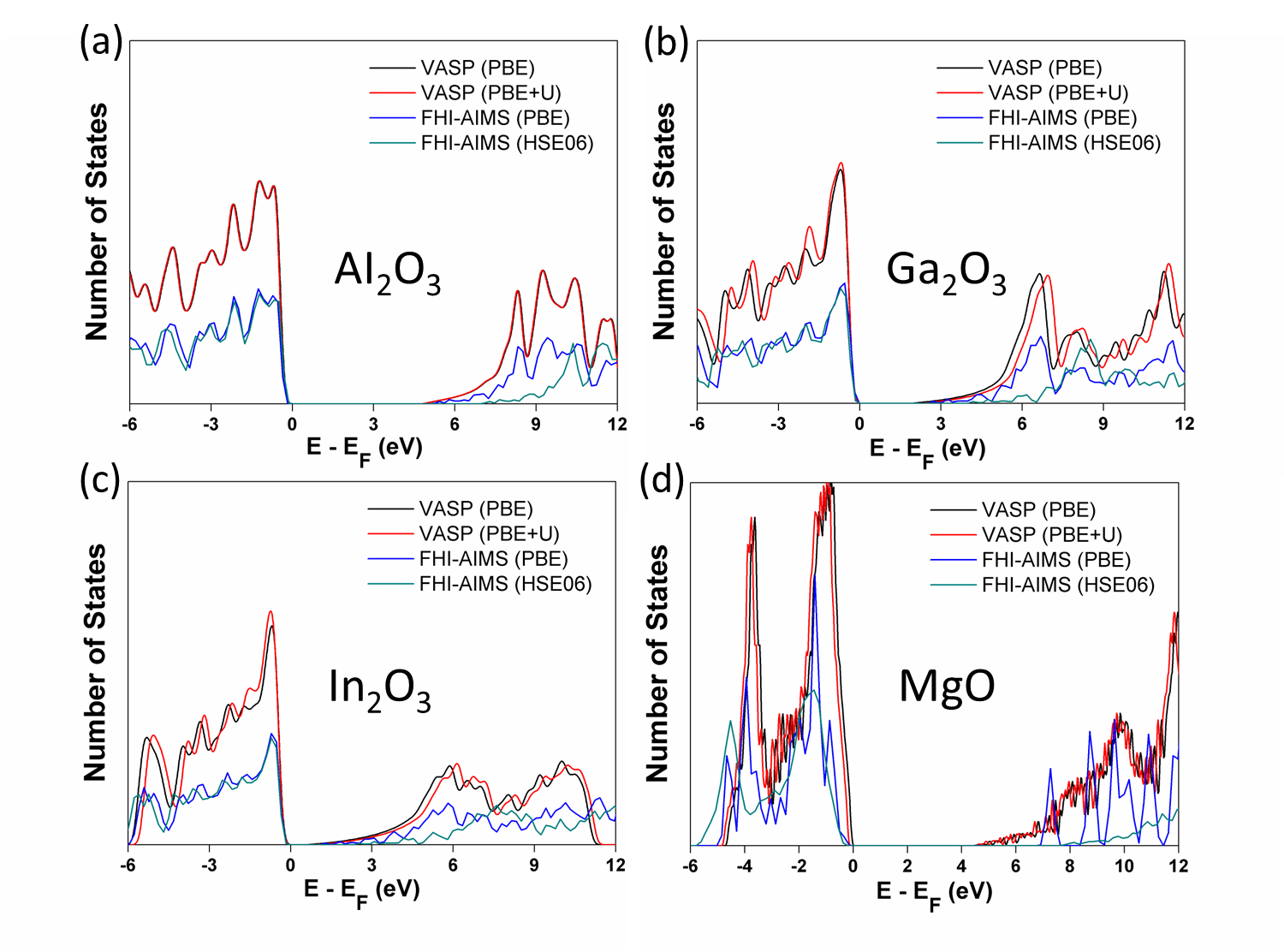
**

**Figure S7.** Relationship between $E_{O_{vac}}$ , band gap and lattice parameters. The unit of *x* and *y* axis is in Å and eV respectively.


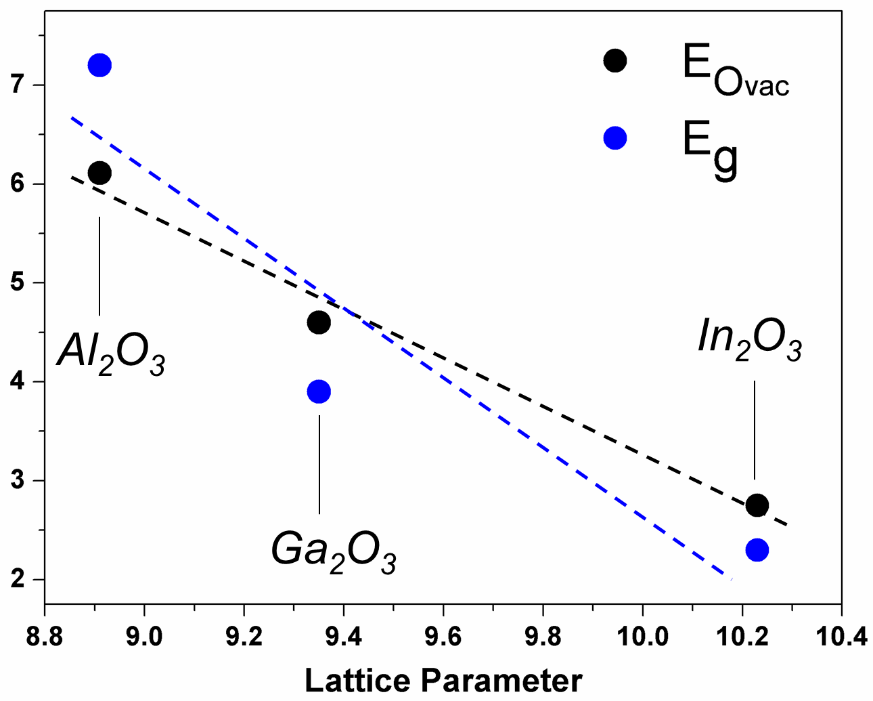

Supplement: Supplementary file 1 — Supplementary file1 (DOCX 2233 KB) [file 894_2024_5957_MOESM1_ESM.docx]
